# Shockwave-Loading-Induced Enhancement of *T*_c_ in Superconducting Bi_2_Sr_2_CaCu_2_O_8+δ_

**DOI:** 10.1038/s41598-017-06887-5

**Published:** 2017-07-27

**Authors:** Tiansheng Liu, Chao He, Fengying Wang, Yingbin Liu, Xiaoxiang Xi, Ruidan Zhong, Genda Gu

**Affiliations:** 1 School of Chemical Engineering and Environment, North University of China, Taiyuan, 030051 China; 2 Photon Sciences, Brookhaven National Laboratory, Upton, NY 11973–5000 USA; 3 Condensed Matter Physics and Materials Science Department, Brookhaven National Laboratory, Upton, NY 11973–5000 USA

## Abstract

We report a shockwave method for altering the properties of the superconductor material Bi_2_Sr_2_CaCu_2_O_8+δ_ (Bi2212). We find that the superconducting transition temperature (*T*_c_) increases from 84 K for the pristine sample to 94 K for the sample treated at a temperature and pressure of ~1200 K and ~31 GPa, respectively. X-ray diffraction and transmission electron microscopy characterizations indicate that this *T*_c_ enhancement arises from a phase transition from pristine Bi2212 to a mixture of superconducting Bi2212 and semiconducting Bi_2_Sr_2_CuO_6+δ_ (Bi2201) during the shockwave treatment. The shockwave-treated sample exhibits n-type semiconductor properties (with an on-off ratio ~5), in contrast to the pure metallic pristine sample. Our study offers an alternative route for modifying the superconducting properties via a shockwave treatment. Furthermore, this method may provide a new approach for studying other temperature- and pressure-sensitive materials.

## Introduction

Bismuth-based copper oxide is one of the most practical high-temperature superconducting material families. It consists of three main phases: Bi_2_Sr_2_CuO_6_ (Bi2201), Bi_2_Sr_2_CaCu_2_O_8_ (Bi2212) and Bi_2_Sr_2_Ca_2_Cu_3_O_10_ (Bi2223)^1–3^. Since their discovery in 1986, these cuprates have been viewed as exotic materials^4^ and have been well-studied by surface-sensitive measurements such as angle-resolved photoemission spectroscopy (ARPES) and scanning tunneling microscopy/spectroscopy (STM/STS)^5–9^. Among known superconductors in the large family of 2-D layered materials, Bi2212 exhibits a high superconducting transition temperature (*T*_c_). Many different methods, including doping^10^, high pressure^11^ and controlling the size of the superconductor at the atomic level^12^, have been investigated to improve the superconducting transition temperature and modify the properties of high-temperature superconductors. Among these approaches, the high-temperature-and-high-pressure technique has attracted much attention as an effective method for the synthesis of new materials^13^ and for tuning the properties of known materials. Many materials become superconducting under a certain pressure, including Bi_2_Te_3_, Sb_2_Se_3_, H_2_S, and WTe_2_^14–16^, motivating many researchers to direct their attention to pressure-induced superconductors. However, most of these efforts have focused on the exertion of hydrostatic pressure^14, 15, 17–21^ and element substitution^3, 22–25^. Direct loading on superconductor materials with dynamic high temperature and high pressure has not been reported to date.

An explosive is a high-energy material that can generate a large amount of heat and gas instantaneously, with the energy of the explosion generating high temperatures and pressures in the form of a shockwave. The synthesis of diamond through the method of explosion has demonstrated that the crystal structure of graphite can be converted into diamond under blasting^26–29^. This method does not rely on sophisticated equipment, and the treatment cycle is short and inexpensive, encouraging us to treat high-temperature superconductors with explosion energy.

In this work, we report a novel method for modifying the properties of the Bi2212 superconductor using a homemade explosive device (Fig. S1a). After the explosion, several techniques were used in combination to characterize the treated Bi2212 sample, including XRD, transmission electron microscopy (TEM), and superconducting quantum interference device (SQUID) magnetometry. We found that the original Bi2212 was transformed into a mixture of the superconducting Bi2212 phase and the semiconducting Bi2201 phase, with an increase in *T*_c_ from 84 to 94 K. Additionally, n-type semiconductor properties of the shockwave-treated samples were demonstrated through measurements of a field-effect transistor device whose on-off ratio can reach 5. Our study not only presents a novel method for modifying the properties of high-temperature superconductors but also paves the way for a new approach to investigating other materials.

## Results

### Phase composition analysis

**Figure 1:**
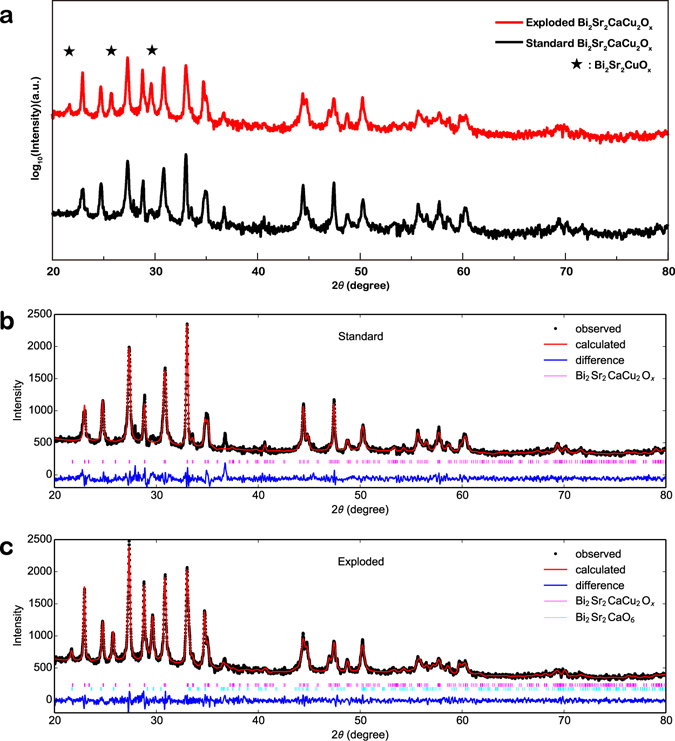
XRD pattern and Rietveld refinement of pristine and shockwave-treated samples. (**a**) Original XRD pattern of the pristine (black line) and the treated (red line) samples; the asterisks represent the newly produced phase. Corresponding Rietveld refinement results for the pristine sample (**b**) and the treated sample (**c**). Black dots are experimental data, red curves are fitting results, blue curves represent the difference between experimental and fitting results, and short vertical lines are the predicted positions of the diffraction peaks.

XRD and TEM measurements were performed to determine the change in chemical composition and structure of the samples induced by the shockwave treatment. Figure 1a shows the XRD pattern of pristine (black line) and treated (red line) samples, with the new produced phase Bi2201 indicated by asterisks. Based on the original data, quantitative phase analysis (QPA) was performed by Rietveld refinement^30–32^ using the GSAS + EXPGUI program; the fitting results are presented in Fig. 1b and c. As shown in Fig. 1b, no distinguishable difference is observed between the calculated and observed results. The final refined structural parameters of the pristine Bi2212 phase were calculated (*a* = 5.41 Å, *b* = 5.41 Å, *c* = 30.86 Å) and are consistent with the previously reported values^33^. After the shockwave treatment, the lattice constants of Bi2212 did not show obvious changes (*a* = 5.41 Å, *b* = 5.41 Å, *c* = 30.93 Å), whereas the lattice constants of Bi2201 (*a* = 5.39 Å, *b* = 5.37 Å, *c* = 20.10 Å) differed substantially from the values for Bi2212. Additionally, the weight fractions of the two phases were found to be 78.70% for Bi2212 and 21.30% for Bi2201. The XRD pattern analysis confirmed our previous prediction that some of the pristine-phase Bi2212 transformed into Bi2201 under the shockwave loading.

**Figure 2:**
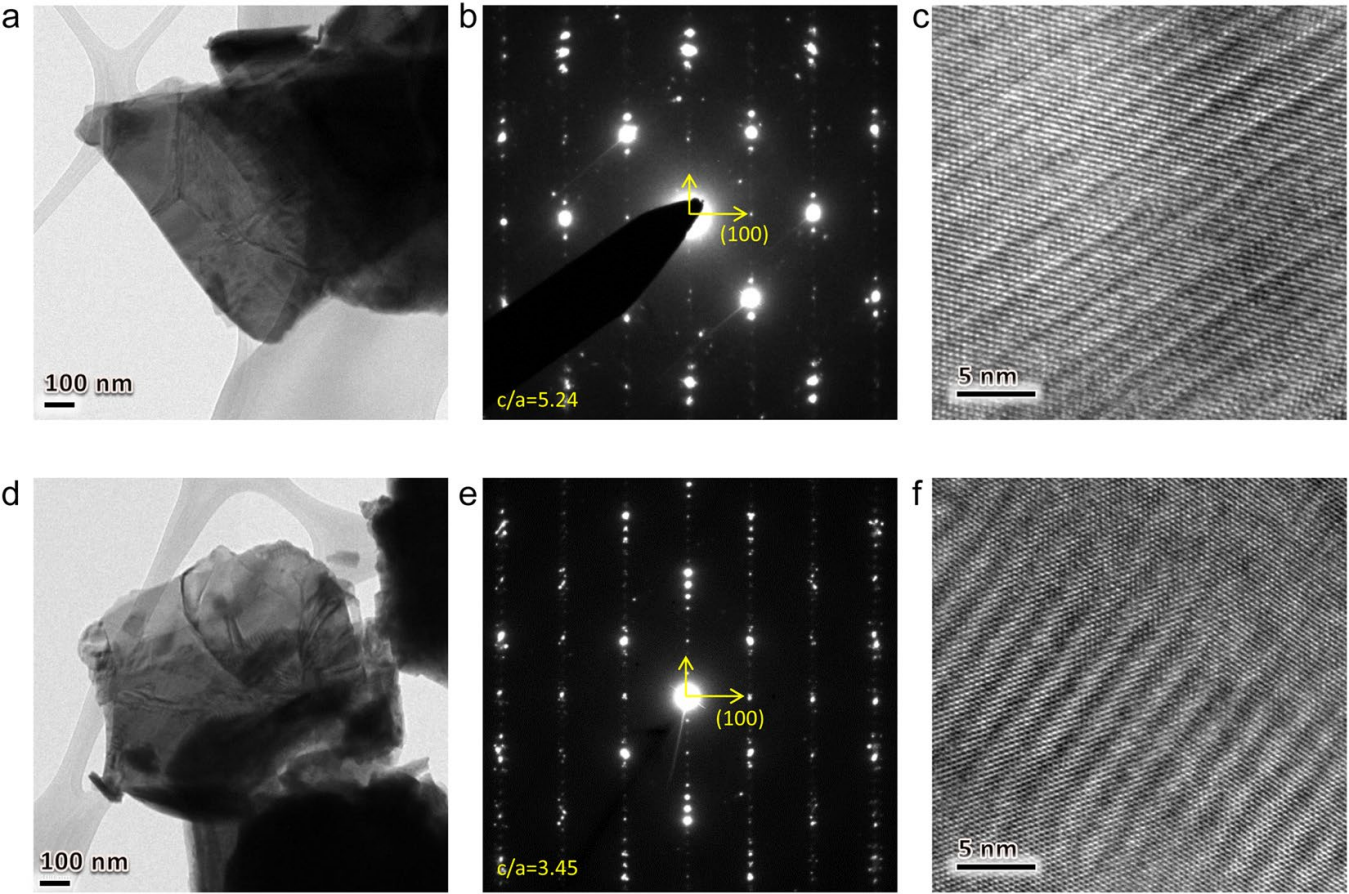
TEM characterization of the pristine and shockwave-treated samples. (**a**) TEM image of a pristine particle. (**b**) Corresponding selected-area electron diffraction (SAED) pattern. The lattice ratio *c*/*a* determined from the SAED is ~5.24. (**c**) Corresponding high-resolution TEM image. The lattice ratio *c*/*a* measured from the fast Fourier transform (FFT) pattern based on a high-resolution TEM image is ~5.36. (**d**) TEM image of a treated particle. (**e**) SAED pattern of the same treated particle. The measured lattice ratio *c*/*a* for this particle is ~3.45. (**f**) High-resolution TEM image of the same treated particle. The lattice ratio *c*/*a* determined from the fast Fourier transform (FFT) pattern is ~3.67.

The phase transition induced by the shockwave treatment was also confirmed by the TEM observations, as illustrated in Fig. 2a–f. In the selected-area electron diffraction (SAED) pattern in Fig. 2b, we find that the lattice ratio *c*/*a* for the pristine sample is ~5.24. Examination of the fast Fourier transform (FFT) pattern based on high-resolution TEM image in Fig. 2c shows that the *c*/*a* ratio is ~5.36, which is close to the value obtained from our XRD data (30.93/5.36 = 5.77). After the explosion, *c*/*a* ratio values of ~3.45 and ~3.67 were obtained from the SAED (Fig. 2e) and the FFT (Fig. 2f), respectively; these values are much smaller than those of the bulk-like Bi2201 superconductor. Note that both the XRD and the TEM analyses indicate that a new phase was produced after the shockwave treatment, which had a short lattice constant *c* of 20.10 Å.

### Magnetization and resistivity measurements

**Figure 3:**
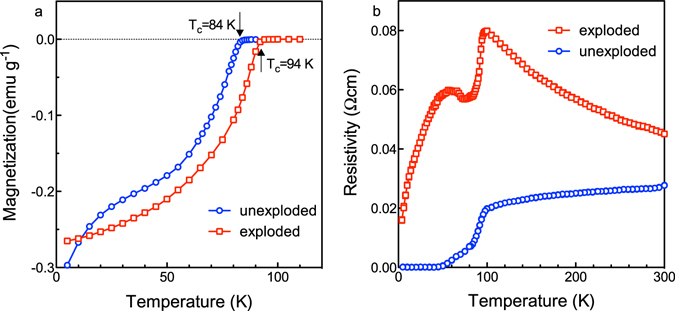
Flux-exclusion and electrical measurements of the pristine and shockwave-treated samples. (**a**) Magnetization-temperature curves of Bi2212 before and after the shockwave treatment. (**b**) Resistivity-temperature curves. Blue and red lines represent the relationships between resistivity and temperature of the pristine and treated samples, respectively.

Figure 3a shows the magnetization-temperature (M-T) curves of the pristine and treated samples. A comparison of the two curves shows that the magnetic susceptibility changes unevenly with temperature before the shockwave treatment, where the slope between 15 K and 60 K is smaller than the slopes corresponding to the 0–15 K and 60–84 K ranges. By contrast, for the treated sample, the slope changes smoothly with temperature, indicating that the internal crystalline phase transformed uniformly under the influence of high temperature and high pressure caused by the shockwave treatment. Interestingly, the critical temperature *T*_c_ of Bi2212 increased from 84 K (pristine) to 94 K (treated), indicating that the shockwave treatment did not destroy the intrinsic properties of Bi2212 and even increased the *T*_c_ by 10 K.

The temperature dependence of the resistivity is shown in Fig. 3b. For the pristine Bi2212 sample, a *T*_c_ onset of 100 K was observed, in agreement with previously reported values^21, 22^, and the material exhibited metallic-like conductivity at temperatures greater than 100 K. Additionally, a *T*_c_ of 84 K and zero resistivity at ~55 K are also evident from the blue curve. For the treated sample composed of a combination of Bi2212 and Bi2201, the resistivity did not decrease to zero. The resistivity was 0.016 Ω.cm at approximately 0 K and showed two peaks at ~55 K (0.06 Ωcm) and ~100 K (0.08 Ωcm). The residual resistivity may be due to the presence of both the superconductor and semiconductor phases in the sample. The *T*_c_ is 94 K, consistent with the M-T curves. Notably, the resistivity decreased very rapidly at temperatures greater than 100 K, and the material exhibited semiconductor behavior.

### Field-effect transistor measurements

**Figure 4:**
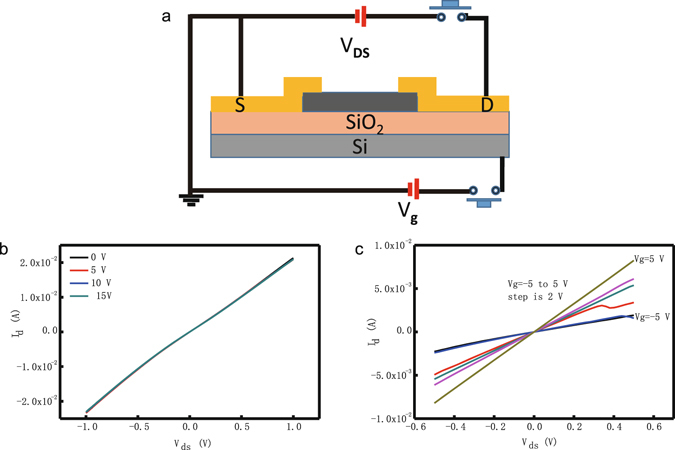
I_DS_–V_DS_ characteristics of the pristine and shockwave-treated samples measured at different V_GS_. (**a**) The FET device. (**b**) I_DS_–V_DS_ curves of the original powder device. The gate voltage changes from 0 V to 15 V in steps of 5 V. (**c**) I_DS_–V_DS_ curves of the device fabricated using shockwave-treated material. The gate voltage changes from -5 V to 5 V in steps of 2 V.

Electrical transport measurements were performed to elucidate the semiconducting properties of the newly produced phase Bi2201 after the shockwave treatment. First, we collected current-voltage (I-V) curves for the device fabricated using pristine Bi2212. Figure 4b clearly shows that the I_DS_–V_DS_ curve is rather symmetric and linear, indicating the presence of ideal ohmic contacts between the metal contact and the Bi2212 powder channels. The back gate voltage V_G_ was applied to Si, but the I_DS_ did not show an obvious change as the gate voltage was varied from 0 V to 25 V in steps of 5 V. The current was approximately 0.02 A when the source-drain voltage was applied at ± 1 V. These results clearly show that the original superconductor powder only exhibited metallic properties, with no obvious semiconductor characteristics. The treated sample was measured under the same conditions; the results are shown in Fig. 4c. The I_DS_–V_DS_ curve is also symmetric and linear, but the I_DS_ could be efficiently tuned by the back gate. The current changed from 1.5 mA to 8.2 mA as the gate voltage was changed from −5 V to 5 V, demonstrating an on-off ratio greater than 5. Although the on-off ratio value is not high (<1000), semiconducting behavior is clearly demonstrated in the I-V curve. Further efforts to enhance the on-off ratio should focus on purifying the sample or controlling the thickness of the device so that the electric field can effectively tune the measured current.

## Discussion

In summary, we have used a new shockwave method to modify some of the properties of polycrystalline superconducting oxide Bi2212. A fraction of the Bi2212 phase was transformed into the semiconducting Bi2201 phase under high temperature and pressure; trace amounts of other phases such as the Bi2223 superconducting phase were also present. The mechanism proposed for the formation of Bi2201 is the compression in the *c*-axis direction, resulting in a short *c* dimension of 20.10 Å in Bi2201 versus 30.93 Å in Bi2212. The diamagnetism critical temperature of the sample was improved from 84 K to 94 K. It is possible that a change in the oxygen content of the Bi2212 contributed to the enhancement of the transition temperature. The shockwave-treated sample exhibits apparent n-type semiconductor behavior, with an on-off ratio a high as 5; by contrast, the pristine sample shows obvious metallic-like properties.

## Methods

### Sample preparation

High purity (99.9%) powders of Bi_2_O_3_, Sr_2_O_3_, CaCO_3_, and CuO were used to prepare the samples in this work. These materials were weighed in the appropriate proportions according to nominal composition Bi_2.1_Sr_1.9_CaCu_2_O_8+x_ and then mixed and calcined at 1023 K, 1073 K, and 1103 K. The homogenous mixture was pressed into sticks using static pressure and then calcined at 1123 K. Finally, the sticks were milled into powders for the subsequent shockwave treatment.

### Shockwave treatment

The explosive used in this experiment was octogen, with the chemical formula C_4_H_8_N_8_O_8_. Bi2212 powders and high-energy explosives with ${{\rm{\rho }}}_{0}$ = 1.80 g/cm^3^ were pressed into cylinders (Fig. S1b,c) using a cylindrical mold. To achieve a high actuating pressure, high temperature and long function time, we designed a special explosive device (Fig. S1a) consisting of a fully sealed internal and external tank. Thermal initiation of detonation (the assembled installation was placed into a heating furnace and heated to explosion) was adopted to allow the explosion occur synchronously in both directions. The explosion action was divided into three stages: first, direct action of detonation, second, multiple reflection of the blast wave and third, action of the explosive products (gas). The detonation velocity was approximately 8310 m/s. The maximum effective detonation pressure calculated by one-dimensional steady C-J detonation theory ($P=\frac{1}{4}{\rho }_{0}{D}^{2}$) was 31.4 GPa, which is consistent with the end pressure of powder grain Bi2212 measured by Manganin gauge. After the shockwave treatment, the explosive device was naturally cooled to room temperature in the furnace by turning the power off.

### Characterization and analysis

XRD measurements were performed at Brookhaven National Laboratory. Rietveld refinement of the XRD diffraction patterns were performed using the GSAS + EXPGUI program in the space groups *A*2*aa* and *Amaa* ^33, 34^. TEM observations were performed using a JEOL2100F microscope operated at 200 kV. A SQUID magnetometer was used for M(T) and *T*_c_ measurements at temperatures in the 2–100 K range, the standard four-probe method was used for R(T) measurements in the 4–270 K temperature range, and MPM was used for *T*_c_ measurements in a magnetic field of 20 G. To compare the properties of the sample before and after the explosion, FET devices were fabricated on SiO_2_/Si substrates. The powder was pressed into thin slices (4 × 1 × 1 mm).

### Data Availability

The datasets generated during and/or analysed during the current study are available from the corresponding author on reasonable request.

## Electronic supplementary material

Supplementary Information
