# Supplementary material for: Shockwave-Loading-Induced Enhancement of Tc in Superconducting Bi2Sr2CaCu2O8+δ
Source: Sci Rep. 2017 Jul 27;7:6710. doi: 10.1038/s41598-017-06887-5 (PMC5532231; doi:10.1038/s41598-017-06887-5)
Supplement: Supplementary file 1 — Supplementary Information [file 41598_2017_6887_MOESM1_ESM.pdf]

Supplementary Information for:

Shockwave-Loading-Induced Enhancement of T_c in Superconducting $Bi_2Sr_2CaCu_2O_{8+\delta}$

Tiansheng Liu[1*], Chao He[1], Fengying Wang[1], Yingbin Liu[1], Xiaoxiang Xi[2], Ruidan Zhong[3], and Genda Gu[3*]

[1]School of Chemical Engineering and Environment, North University of China, 030051, China

[2]Photon Sciences, Brookhaven National Laboratory, Upton, NY 11973–5000, USA

[3]Condensed Matter Physics and Materials Science Department, Brookhaven National Laboratory, Upton, NY 11973–5000, USA

*Email: liutsh66@sina.com.cn; ggu@bnl.gov

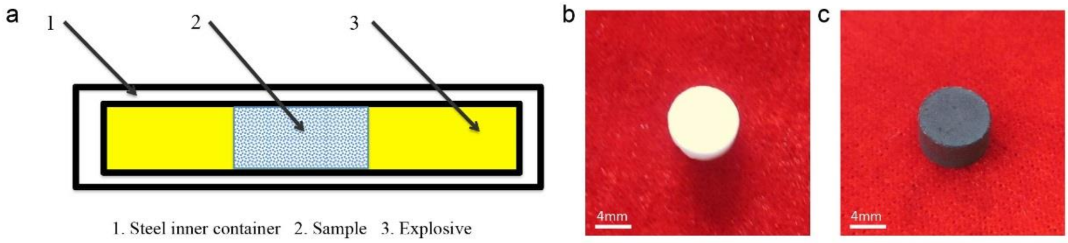

Figure S1. Schematic of the explosive device (a) and photographs of the corresponding explosive (b) and sample (c).
